# A Winding Road: Alzheimer’s Disease Increases Circuitous Functional Connectivity Pathways

**DOI:** 10.3389/fncom.2015.00140

**Published:** 2015-11-18

**Authors:** John Suckling, Tiago Simas, Shayanti Chattopadhyay, Roger Tait, Li Su, Guy Williams, James B. Rowe, John T. O’Brien

**Affiliations:** ^1^Department of Psychiatry, University of CambridgeCambridge, UK; ^2^Cambridge and Peterborough Foundation NHS TrustCambridge, UK; ^3^MRC/Wellcome Trust Behavioural and Clinical Neuroscience Institute, University of CambridgeCambridge, UK; ^4^Department of Clinical Neurosciences, University of CambridgeCambridge, UK; ^5^Wolfson Brain Imaging Centre, University of CambridgeCambridge, UK

**Keywords:** neuroimaging, Alzheimer disease, functional connectivity, transitivity, semi metricity, connectome

## Abstract

Neuroimaging has been successful in characterizing the pattern of cerebral atrophy that accompanies the progression of Alzheimer’s disease (AD). Examination of functional connectivity, the strength of signal synchronicity between brain regions, has gathered pace as another way of understanding changes to the brain that are associated with AD. It appears to have good sensitivity and detect effects that precede cognitive decline, and thus offers the possibility to understand the neurobiology of the disease in its earliest phases. However, functional connectivity analyzes to date generally consider only the strongest connections, with weaker links ignored. This proof-of-concept study compared patients with mild-to-moderate AD (*N* = 11) and matched control individuals (*N* = 12) based on functional connectivities derived from blood-oxygenation level dependent (BOLD) sensitive functional MRI acquired during resting wakefulness. All positive connectivities irrespective of their strength were included. Transitive closures of the resulting connectome were calculated that classified connections as either direct or indirect. Between-group differences in the proportion of indirect paths were observed. In AD, there was broadly increased indirect connectivity across greater spatial distances. Furthermore, the indirect pathways in AD had greater between-subject topological variance than controls. The prevailing characterization of AD as being a disconnection syndrome is refined by the observation that direct links between regions that are impaired are perhaps replaced by an increase in indirect functional pathways that is only detectable through inclusion of connections across the entire range of connection strengths.

## Introduction

Within neuroimaging, the integrationist perspective derived from measures of the blood oxygenation-level dependent (BOLD) signal synchronicity between regions has become the primary tool for understanding the functional organization of the brain with fMRI. So much so, that the annual rate of publications on functional integration now exceeds that on functional localization (i.e., task-induced activation; Friston, 2011). This approach has been highly successful in providing a new substrate and vocabulary to express both the principles of healthy brain organization, and the generalised changes to its key elements that are associated with disorder (Lynall et al., 2010).

Evidence from structural neuroimaging over the last two decades has provided a convergent picture of the changes to gray matter that occur before, during and after a formal diagnosis of Alzheimer’s disease (AD; Frisoni et al., 2010). Beginning in the medial temporal lobe, and in particular the hippocampus, atrophy continues in temporal, parietal and frontal cortices (Scheltens et al., 1992; Frisoni et al., 2010; Matsuda, 2013; Wang et al., 2015a) closely following the neuropathological progression (tau pathology) of the disease (Braak and Braak, 1991) and the course of decline in cognition (Frisoni et al., 2010). Investigations of functional connectivity in AD are still relatively immature, nevertheless there is an emerging broad narrative.

A major discovery of the integrationist approach has been the default mode network (DMN), consisting of the medial prefrontal cortex, posterior cingulate cortex, precuneus, anterior cingulate cortex, and parietal cortex, and activated when the brain is not otherwise engaged in cognitively demanding processing. These regions are highly connected with each other and overlap with brain regions—hubs—that are disproportionately globally connected. Evidence for the involvement of the DMN in AD is extensive and compelling (Mevel et al., 2011; Hafkemeijer et al., 2012; Vemuri et al., 2012). The DMN largely coincides with concentrations of amyloid protein deposition in patients with AD (Buckner et al., 2009), connectivity changes within it are correlated with progression of the disease (Damoiseaux et al., 2012), and its structural pathology (Buckner et al., 2005). Furthermore, the DMN appears unable to rapidly adapt to cognitive demand that occurs at the earliest stages of the disease (Rombouts et al., 2005). However, the DMN is not necessarily the only network involved in AD. Other networks have also been implicated, particularly the salience network (Zhou et al., 2010; consisting of the anterior cingulate cortex and medial temporal regions, including the insula) and the sensorimotor network (Dipasquale et al., 2015; pre- and post-central gyri). The possibility that widespread disconnectivity characterizes AD motivates a more global investigation of brain (dis)organization.

### Strong and Weak Connections of the Functional Connectome

Concisely describing the myriad functional connections of the brain is predominantly undertaken with graph theoretical approaches. To generate a graphical object suitable for characterization by a small number of parameters, edges (often defined by the bivariate correlation of BOLD time-series between two brain regions) that connect nodes (the brain regions themselves) are necessarily sparse and frequently binary; that is, edges are either present or absent. This is generally achieved by a threshold on the correlation coefficients, also known as the cost, that may be followed by algorithms that ensure all remaining nodes within the connectome are connected by, for example, reinstating “missing” edges at minimum cost (Ciftci, 2011). Once constructed, parameters of the resulting connectome can be derived. This has been a powerful approach that has identified key features of the functional connectome such as hubs and its “small-world” topology (Bassett and Bullmore, 2006); that is, a graph with a small average minimum number of edges that connect any two nodes, but with relatively greater local connectivity.

As with all models, several assumptions constrain interpretation of binary connectomes. In particular, thresholding the matrix of values representing the connectivity between regions leads to a focus on the clique of edges present at high costs, typically only 5–20% the total number. From the resulting connectome the implicit assumption is that information flow occurs through these connections and often preferentially along the shortest path, typified by the systematic variation of cost to generate small-world networks that are particularly efficient in this regard.

In general, the role of weak edges has been underrepresented to date in the integrationist perspective of the brain. In contrast, sociological theory has long recognized that weak links play a vital role in the distribution of information through a network of friends and acquaintances that can be represented as a graph (Granovetter, 1973, 1983). In fact, it can be demonstrated theoretically and empirically that unless a node is isolated from all other nodes but one, strong edges are highly unlikely to uniquely connect two nodes. In other words, there will almost always be an alternate path between any two strongly connected nodes. However, this is not the case for weak edges which can “bridge” the span between strongly connected local nodes. Indeed, in Milgram’s original experiment of social connections (Travers and Milgram, 1969), both strong and weak links were necessary for the “six degrees of separation” that has subsequently entered into common parlance.

The integrationist approach, and in particular the use of graph theory as its formal framework, has drawn inspiration from a wide variety of real-world examples. Yet in relation to how information traverses the network to arrive at its intended destination, whether the sender has available to them a map of the overall structure is important to the strategies deployed. If so, then it is possible to plot the shortest-paths and use this knowledge to efficiently transport whatever is carried on the network; “information” in the case of the brain. If not, then transport will rely on the distributed broadcast of information, for example via random walk (Noh and Rieger, 2004), where edges of all strengths play a role and a more efficient route involving “weak” edges may not be the most direct.

For example, a passenger travelling on the airline network has access to a map with which to plan, in advance, the most parsimonious route. Conversely, a sender asked to email a message via friends and acquaintances to a recipient chosen at random is likely to be ignorant of the overall social network, and will instead choose a more egalitarian approach with weak links over-represented in the chains (Dodds et al., 2003).

Making the assumption that the brain does not have an overall, instantaneous representation of its own connectome, an assertion given credence by the temporal variance of its edges (Zalesky et al., 2014), the transfer of information may well involve the entire, fully-connected connectome taking advantage of the proletariat of weak edges. This ensemble of edges may support several types of trajectory that information may follow (shortest paths, paths with redundancy, or paths that retrace edges) and the manner in which the information is disseminated (as a single item, or duplicated in serial or in parallel; Borgatti, 2005). Interestingly, global firing of neurons in an *in vitro* culture is controlled by a pulse of activity that is randomly nucleated and that rapidly propagates throughout the network (Orlandi et al., 2013), underlining the importance of broadcast dynamics.

One way of investigating this complex network is through the topology of circuitous functional connections that form a stronger path than that offered by the edge that directly links any two nodes. Formally, this is known as a transitivity violation which occurs if the distance of an indirect path between two nodes is less than the distance of the direct path between them. This type of network is called *semi-metric* and is embedded in a non-metric topology (Simas and Rocha, 2015). Generally, any weighted network will have some degree of semi-metricity. In recent work, we have shown that in many types of real-world networks the levels of semi-metricity are high (Rocha, 2002; Simas and Rocha, 2012, 2015); that is, networks have a high degree of redundancy or increased sharing of information amongst communities.

### Characterisation of AD with the Functional Connectome

Application of the functional connectome in cross-sectional studies of AD has led to a mixed picture (Tijms et al., 2013). There is a consensus that the binary human functional connectome is small-world. However, whilst case-control studies of AD demonstrate alterations to this configuration, the current weight of evidence does not yet strongly indicate whether the changes observed are towards a more disordered, random graph (Sanz-Arigita et al., 2010) or a more ordered, lattice graph (Zhao et al., 2012).

Weighted networks incorporate continuous measures of functional connectivity into the analysis. For connectivities that are thresholded to generate the network, equations characterizing their topologies can be adapted to include the original values, as well as other weightings. This approach has been undertaken on a sample of AD patients compared to controls (Liu et al., 2014). Although local clustering was unchanged, the mean anatomic distance across all edges in the graph was significantly smaller in AD. Furthermore, cognitive impairment was associated with reduced connectivity in long-range connections, in particular.

In general there appears to be strong evidence that disruptions to functional connectivity accompany the well-characterized patterns of cerebral atrophy. The direction of these disruptions is less clear. This is a feature not only of comparisons of networks derived from BOLD sensitive fMRI, but also across imaging modalities that measure brain structure and function from diverse physical and physiological phenomena such as structural MRI, electroencephalography and magnetoencephalography (Tijms et al., 2013).

This article investigates the case-control differences in a small sample of patients with AD and unaffected controls with an integrationist approach to the BOLD-sensitive fMRI acquired whilst participants were at wakeful rest (i.e., without any specific stimuli). The weighted functional connectomes derived were compared by metrics that assess the prevalence of indirect functional connections.

This approach has not previously been applied to data acquired from patients with neurodegenerative disorders and thus it is difficult to hypothesise, *a priori*, the possible outcome of the analysis. Thus, an exploratory whole-brain strategy was adopted first statistical testing scalar metrics of semi-metricity in regions-of-interest, and then qualitatively displaying the distribution of the between-group differences in semi-metric edges.

## Materials and Methods

### Participant Recruitment

Eleven participants with a diagnosis of probable AD based on National Institute on Aging criteria (McKhann et al., 2011), were recruited along with twelve unaffected controls of similar age and gender (Table 1). Participants were recruited from the Neuroimaging of Inflammation in Memory and Other Disorders study (NIMROD), a cross-sectional study of dementia within the UK National Institute for Health Research Cambridge Biomedical Research Unit in Dementia. Participants provided written informed consent. The study was approved by the Cambridge 2 Research Ethics Committee.

**Table 1:** **Demographic characteristics of the groups**.

	AD patients	Controls	Test	*p*-value
Mean age (years) ± SD	67.5 ± 10.1	68.0 ± 5.6	*t*_(df = 21)_ = 0.16	0.87
Mean years of education ± SD	14.3 ± 3.3	14.0 ± 3.0	*t*_(df = 21)_ = 0.21	0.84
Male/Female	8/3	4/8	χ^2^ = 0.10	0.07
MMSE ± SD	24.6 ± 3.3	28.7 ± 1.1	*t*_(df = 21)_ = −4.14	*p* ≪ 0.001

*SD, Standard deviation; df, Degrees of freedom; MMSE, Mini Mental State Examination*.

Inclusion criteria included clinically probable AD supported by abnormal structural MRI (McKhann et al., 2011), age over 50 years, with preservation of mental capacity for consent, absence of contraindications to MRI and no other significant neurological or psychiatric disorder, including depression. All participants underwent a comprehensive assessment including full medical and psychiatric history, cognitive testing and dementia blood screen, a diagnostic screen (comprised of routine hemeatology and biochemistry tests, thyroid function tests, vitamin B12, and folate levels) used to help make the diagnosis of dementia subtype and exclude thyroid and vitamin deficiencies.

### Resting fMRI Data Acquisition

Blood oxygenation level-dependent sensitive imaging data were acquired on a 3T MRI scanner (Magnetom^®^ Verio; Siemens AG, Erlangen, Germany) at the Wolfson Brain Imaging Centre, University of Cambridge, with a multi-echo echo-planar imaging (EPI) sequence (Poser et al., 2006) using the following parameters: repetition time, TR = 2430 ms; echo times TE = 13, 31 and 48 ms; flip angle = 90^o^; reduction of single volume acquisition time with sensitivity-encoded single-shot EPI (SENSE) by a factor of 2; acquisition matrix = 64 × 64; number of slices = 38; field-of-view, FOV = 240 mm × 240 mm and slice thickness = 3.8 mm (i.e., voxel size = 3.8 × 3.8 × 3.8 mm^3^); acquisition time = 11 min 9 s. During acquisition, participants were asked to lie quietly with their eyes closed.

To test for between-group differences in head motion, independent *t*-tests (assuming unequal variances) were undertaken of mean DVARS, the average root mean square variance across all brain voxels of volume-to-volume difference in percent signal change, and the final relative to initial image displacement (translations and rotations about orthogonal axes). These metrics represent rapid and slow rates of head motion, respectively.

### Structural MRI Data Acquisition

Brain structure was imaged with a T1-weighted volumetric magnetization-prepared-rapid acquisition gradient-echo (MP-RAGE) sequence using the parameters: TR = 2300 ms; TE = 2.98 ms; inversion recovery time = 900 ms; flip angle = 9°; acquisition matrix = 256 × 240; number of slices = 176; FOV = 256 mm × 240 mm and slice thickness = 1.0 mm (i.e., voxel size = 1.0 × 1.0 × 1.0 mm^3^); acquisition time = 9 min 14 s. All MRIs were reviewed by a neuroradiologist to exclude space occupying lesions and ensure that scans were consistent with AD, but were not formally rated on scales for cortical or hippocampal atrophy.

Whole brain voxel based morphometry (VBM) tested for anatomical differences in gray matter volume (GMV) between patients and controls. Voxelwise estimates of GMV from the T1-weighted structural MRI images were calculated using an optimized VBM protocol (Good et al., 2001) using version 5 of FSLVBM (Smith et al., 2004) from the Functional Magnetic Resonance Imaging of the Brian software library (FSL)^1^.

First, the brain was identified and masked in the T1-weighted MRI (Smith, 2002), followed by segmentation into gray and white matter volume maps using FAST (Zhang et al., 2001). GMV maps were initially aligned to the Montreal Neurological Institute (MNI) standard space atlas by affine registration, followed by non-linear registration. Modulation controlled for changes to voxel morphology during registration. The modulated GMV maps were then smoothed with an isotropic Gaussian kernel with a variance of 3 mm.

Between-group differences in GMV were tested by *t*-test and regression of the general linear model. Significance testing was undertaken using threshold-free cluster enhancement (Smith and Nichols, 2009) with the statistical threshold for significance set at *p* < 0.05, Family-Wise Error (FWE) corrected for multiple comparisons.

### Resting fMRI Preprocessing

The multi-echo EPI acquisitions were first converted to a single value with near optimal contrast-to-noise ratio using a weighting scheme on acquisitions at each TE (Poser et al., 2006). Imaging data were then pre-processed to account for head motion (Patel et al., 2014). In brief, the first four volumes of each resting state data set were removed to eliminate the non-equilibrium effects of magnetization leaving 256 volumes for analysis. Preprocessing steps then included slice-time correction, temporal despiking, high pass frequency filtering above 0.02 Hz, co-registration to the accompanying structural scan, spatial normalization to the standard space of the Montreal Neurological Institute (MNI) template, and spatial smoothing (6-mm full width at half maximum Gaussian kernel). This toolbox (BrainWavelet Toolbox)^2^ corrects for motion by regressing out motion parameters, and cerebrospinal fluid (CSF) signal from ventricular regions.

### Semi-Metric Percentages

For each individual the mean time-series was extracted from each of 116 anatomically parcellated regions (i.e., nodes) based on the Eickhoff-Ziles (EZ116) atlas (Eickhoff et al., 2005). The extracted BOLD signals were decomposed into four frequency bands by wavelet transform: scale 1, 0.125–0.25 Hz; scale 2, 0.06–0.125 Hz; scale 3, 0.03–0.06 Hz; scale 4, 0.02–0.03 Hz. Scale two was selected since it represents the wavelet scale where the BOLD signal has previously been strongly correlated with aberrant physiology involved in a range of psychiatric disorders. The strength (i.e., proximity) of a connection between two nodes was the Pearson’s correlation coefficient of the wavelet coefficients.

Pearson’s correlation measures the synchronicity between endogenous BOLD signals. Positive values corresponds to in-phase responses, and negative values for signals in anti-phase. Combining both phenomena in one network is non-trivial since positive and negative values potentially represent different types of connections. For this reason adjacency matrices were constructed from only the non-negative, bivariate correlations. Edges associated with negative correlation were excluded from the subsequent semi-metric analysis; that is, they were considered to represent very large distances.

The shortest path between nodes of a network is an important feature as it is a measure of information exchange between two or more nodes connected by edges with which there is an associated weighting (here, the correlation of BOLD activity during resting wakefulness). It is defined as the route between two nodes that minimizes the sum of the distances of the edges that are traversed. When the shortest path is the direct connection between the nodes it is known as a *metric* edge. Conversely, when the shortest path is circuitous—via additional nodes—it is known as a *semi-metric* edge.

To find the shortest path the proximity can be maximized through transitive closure. The functional connectivities between nodes are first converted to distance relations that describe all the destinations that are reachable from a given node, by the isomorphism:

$$\varphi :d_{\text{ij}}=\frac{1}{w_{\text{ij}}}-1$$

where *w_ij_* is the proximity (i.e., correlation coefficient) and *d_ij_* is the distance between any pair of nodes *i* and *j*. In some cases, the triangle inequality may be violated: *d_ij_* ≥ *d_ik_* + *d_kj_* for some element, *k* (which generalises to transitivity violations involving any number of other nodes). Thus, the shortest path between two elements may not be the direct edge, but rather an indirect path via a number of edges. The shortest direct or indirect path between nodes in the distance graph is calculated by metric closure, using by Johnson’s algorithm (Johnson, 1954), leading to a classification of each path as metric or semi-metric (for more details, see Simas and Rocha, 2015; Simas et al., 2015). These calculations were performed with Matlab (MathWorks, USA).

The semi-metric percentage (SMP) measures the overall level of semi-metric behavior of a network or sub-network, and is defined as the ratio of the number of edges labelled as semi-metric to the total number of edges.

Regional group differences in the SMP were first tested at the whole-brain level, then in a decomposition into sub-graphs for left and right hemispheres, then decomposing hemispheric sub-graphs into lobes and the connections between them (the aggregations of nodes defined using the appropriate levels of the Harvard-Oxford atlas anatomical hierarchy)^3^ to identify regions that contributed to any overall difference in SMP. At each level of the hierarchy, the nodes of graph remained the same and defined by the EZ116 atlas. These tests would not be independent, and thus a scheme was adopted whereby pursuance of statistical testing was predicated on the significance (i.e., *p* < 0.05, two-tailed) of the test in the preceding level in the hierarchy. SMPs from between-lobe, within-hemisphere edges and between-hemisphere edges were tested as measures of more long-range connections. Between-group differences in SMP were also ranked in order of the average Euclidean distance between nodes in the sub-graphs from which they were calculated.

The between-group differences on the whole-brain connectome were also assessed by comparison of the shortest paths following transitive closure. To achieve this, the proximity graphs derived for each individual in each group were aggregated into a multilayer network by embedding them in a distance space using a distance closure, which is isomorphic to a transitive closure in proximity space. During this embedding, semi-metric edges undergo a high degree of distortion. Thus, large differences when taking the absolute value after subtracting the embedded networks corresponding to two groups identifies differences in semi-metric behavior. Full details of the method have been reported previously (Simas et al., 2015). To observe the overall difference in semi-metric behavior that a node has with all other nodes, the sum of between-group differences associated with that node was averaged and reprojected onto the brain surface.

### Semi-Metric Backbones

Variation across individuals of the semi-metric connectomes was visually assessed by construction of the backbone for each group. Every edge on the individual connectomes was labelled as either metric or semi-metric, using the algorithm described above. An edge on the backbone is displayed with a thickness that denotes the percentage of individuals from the group that have a semi-metric connection at that location. The minimum percentage of individuals required to display an edge in the backbone is arbitrary, but was selected here at >90% for depiction of the key features whilst removing connections not commonly shared across the group and serve only to obscure the visualization. Metric backbones may be constructed, but due to the smaller proportion of metric connections they are sparser and thus not as informative.

## Results

### Sample Demographics

Patients and control groups had non-significant differences in age, gender and years of education, but as expected AD participants had significantly lower mini mental state examination (MMSE) scores (Table 1).

### Between-Group Differences in Head Motion

Between-group differences in mean DVARS were non-significant: *t*_(19.31)_ = 0.81, *p* = 0.42 (mean DVARS: AD = 1.00, controls = 0.82).

Between-group differences in the translations and rotations of the final volume relative to the initial volume were non-significant (*p* ≥ 0.05) for displacements in the *x*-direction (left-right: *t*_(20.88)_ = 2.10, *p* = 0.05), *y*-direction (anterior-posterior: *t*_(11.48)_ = 0.15, *p* = 0.88), and *z*-direction (superior-inferior: *t*_(15.52)_ = 1.75, *p* = 0.10), and rotations about the *x*-axis (*t*_(12.48)_ = 0.36, *p* = 0.73), *y*-axis (*t*_(18.11)_ = 2.01, *p* = 0.06), and *z*-axis (*t*_(10.75)_ = −1.03, *p* = 0.33).

### Between-Group Differences in Brain Structure

An independent *t*-test of the total GMVs was significant (*t*_(21.45)_ = 4.42, *p* = 0.00021). However, a whole-brain, between-group comparison of GMV at *p* < 0.05 (FWE corrected) did not reveal any significant differences. Using a more lenient statistical threshold of *p* < 0.001 uncorrected resulted in significant reductions in GMV in AD patients in areas including temporal and parietal regions (Supplementary Figure 1).

### Between-Group Differences in Semi-Metricity

**Figure 1:**
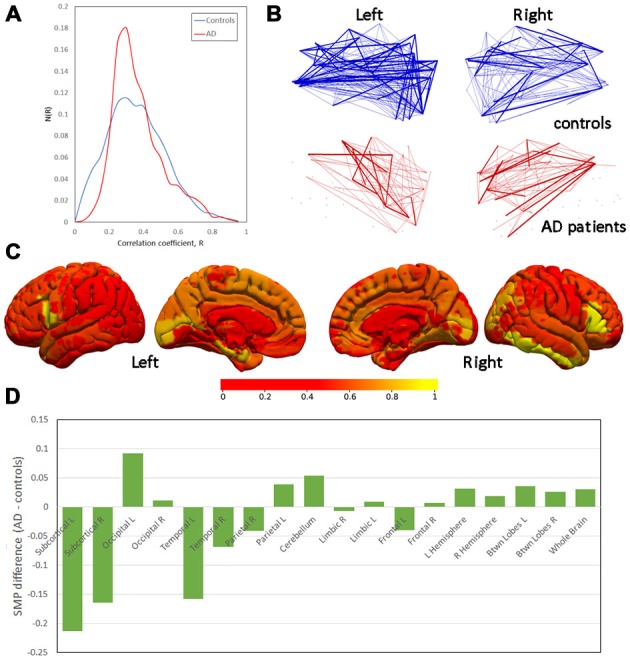
**Differences in semi-metricity. (A)** Histograms of correlation coefficients, R, aggregated across all participants in each group. **(B)** Projections of semi-metric backbones for left and right hemispheres demonstrating the lower number of consistent semi-metric edges in Alzheimer’s disease (AD) patients. **(C)** Between-group differences in the node-averaged semi-metric connections to all other regions (scale normalized for display). **(D)** Between-group differences in semi-metric percentage (SMP) ranked by the mean Euclidean distance between nodes.

Inspection of the histogram of correlation coefficients from all participants in both groups (Figure 1A) indicates an approximately symmetrical decrease in the variation of the distribution corresponding to AD patients, although the maxima are not shifted relative to each other (median R: AD = 0.266; controls = 0.274; mean ± SD: AD = 0.291 ± 0.034; controls = 0.316 ± 0.036). Shapiro-Wilks tests of Normality were highly significant (*p* < 10^−15^) for both groups, and so measures of means and variances were not appropriate characterizations of the distributions.

Edges associated with negative correlation were excluded from semi-metric analysis. An independent samples *t*-test not assuming homogeniety of variance, of the number of negative (i.e., set to zero) correlations was non-significant (*t*_(22)_ = 0.35, *p* = 0.37).

Table 2 gives the details of the between-group differences in SMP, which were significant at the whole-brain level, and increased in AD patients. The right hemisphere SMP was also significantly increased in AD patients as was that associated with inter-hemispheric connections (Table 1). Within the right hemisphere, both the occipital and between-region SMP were significantly increased in AD patients. There was no significant correlation between SMP and GMV (R = −0.32, *p* = 0.14).

**Table 2:** **Whole-brain and the subsequent regional between-group mean differences, 95% confidence intervals and *p*-values in semi-metric percentages (SMP)**.

Region	Difference of means (AD—controls)	Confidence interval (95%)	*p*-value
Whole brain	0.026	0.002, 0.050	0.037*
Left hemisphere	0.007	−0.023, 0.036	0.637
Right hemisphere	0.031	0.005, 0.057	0.022*
Cerebellum	0.039	−0.018, 0.096	0.173
Between-hemispheres	0.03	0.004, 0.056	0.029*
**Left**
Frontal	0.009	−0.049, 0.067	0.749
Parietal	−0.068	−0.174, 0.037	0.191
Occipital	0.092	−0.031, 0.215	0.135
Temporal	−0.158	−0.286, −0.030	0.018*
Limbic	−0.007	−0.110, 0.096	0.888
Subcortical	−0.213	−0.385, −0.041	0.018*
Within-hemisphere,	0.019	−0.007, 0.046	0.146
Between-region
**Right**
Frontal	−0.04	−0.124, 0.043	0.317
Parietal	−0.041	−0.122, 0.040	0.299
Occipital	0.134	0.016, 0.252	0.028*
Temporal	0.011	−0.103, 0.124	0.845
Limbic	0.054	−0.075, 0.183	0.39
Subcortical	−0.164	−0.382, 0.052	0.127
Within-hemisphere,	0.036	0.007, 0.065	0.016*
Between-region

*p < 0.05.

Bearing in mind the absence of group differences in SMP in the left hemisphere (Table 2), the apparent hemispheric symmetry of these results prompted repeated measures *t*-tests (equal variances assumed) for the difference in SMP between left and right hemispheres. These tests were non-significant for all participants in both groups (*t*_(22)_ = −0.81, *p* = 0.43) and AD patients alone (*t*_(10)_ = −1.48, *p* = 0.17). A between-group, independent samples, non-parametric test of the asymmetry index = (r−l)/(r+l) (Geary, 1930), where r and l are the SMPs in the right and left hemispheres respectively, was also non-significant (Mann-Whitney U = 49.00, *p* = 0.20).

A display of the average semi-metric behavior of the nodes broadly recapitulates the results from comparisons of SMPs, but also identifies the bilateral inferior frontal gyri and right inferior temporal gyrus (Figure 1C) as regions of greatest difference. At these nodes, the between-group differences in the shortest indirect paths to all other nodes in the network are greatest, and thus the distortion in distant space needed to achieve the distance closure.

The pattern of between-group differences in SMP as a function of the average distance of the edges within the sub-graphs are shown in Figure 1D. There is a weak apparent trend in change in SMP over the spatial scales of the sub-graphs with greater SMP in controls at short distances and greater SMP in AD patients at longer distances.

Finally, semi-metric backbones (Figure 1B) indicate a widespread decrease in semi-metric edges that are consistent across AD patients. In other words, although indirect paths are generally increased, predominantly in the right hemisphere (Table 2), the edges which are semi-metric vary strongly between AD patients relative to controls.

## Discussion

This study is the first to illustrate the importance of “weak” links in the functional connectome to neuroimaging data from samples of individuals with neurodegenerative disorders, in this case AD. Weak links are important to the overall connectome in that they can form part of indirect paths that are in fact more proximate than the direct path (i.e., the semi-metric paths).

Widespread (i.e., whole-brain) increases in the SMP indicate a change in brain functional connectivity towards more circuitous paths. Whilst regional testing points towards a predominance in the left hemisphere, there was no statistical evidence for any asymmetry in the global effect. A qualitative appreciation of the SMP differences also suggests symmetric changes. There was no strong evidence for any changes in magnitude or sign in semi-metric behavior as a function of geometrical connection distance.

### Global Increases in Indirect Paths are Associated with AD

Cortical atrophy measured with VBM is a robust indicator of disease progression in AD with initial cortical thinning in medial temporal structures later involving limbic, frontal and occipital brain regions, in that sequence (Thompson et al., 2003; Matsuda, 2013). No statistical differences in GMV were observed in this sample of mild-to-moderate AD despite ample evidence that suggests morphological changes can be detected early in the course of the illness (Hirata et al., 2005; Ishii et al., 2005). The implication is that the small samples size (*N* = 11 AD patients) in this study reduced the statistical power below the threshold for detection at *p*-values appropriately controlled for multiple comparisons. This is evidenced by a plausible pattern of GMV reduction seen at uncontrolled thresholds (Supplementary Figure 1). Furthermore, this sample is relatively young and there is evidence that patterns of cerebral atrophy may vary depending the age of onset (Moller et al., 2013). Thus, there may be deviation here from the expected pattern of structural changes observed in older patients.

SMP was sufficiently sensitive to detect case-control differences at the whole-brain level. Significant contributions to the overall differences were localized in the right hemisphere, particularly in the occipital lobe, and in the intra-hemispheric connections between regions. Additionally, the between hemisphere connections also significantly differed. All these changes involved an increase in SMP (Table 2).

Although these regional results suggest asymmetry to the effects, formal statistical testing and a qualitative view of the between-group differences demonstrates a more bilateral, symmetric pattern either in node-average semi-metric connections (Figure 1C) or regional SMPs (Figure 1D). Nevertheless, insufficient power to detect lateralisation due to small sample sizes cannot be disregarded. Ranking the SMP differences by the average between-node distance in the sub-graphs leads to an overall picture of more indirect (semi-metric) paths at greater spatial distances.

### Comparisons with Studies of Strong Functional Connections

The DMN is a network of brain regions in the posterior cingulate, parietal cortex, medial temporal and medial prefrontal cortices associated with atrophy, amyloid deposition and reduced metabolism in AD. A review of functional connectivity studies of the DMN in AD (10) reveals both increases and decreases in functional connectivity between its components. This mixed picture is broadly aligned with the profile of differences observed with this analysis of semi-metricity as well as that obtained with other modalities, particularly with regard to the frequency-dependent change in the sign of the effect at long and short ranges (Stam et al., 2006; Babiloni et al., 2011).

Inferior frontal regions were highlighted as being associated with the greatest between-group difference in terms of their average semi-metric behavior towards all other nodes in the connectome (Figure 1C). These areas are important for inhibition and, in the left hemisphere, language. Reductions in functional correlations have been located in the inferior frontal gryrus as part of disruptions to larger scale brain circuits (Zhou et al., 2013; Zhang et al., 2014).

Long-range, interhemispheric connections have come under scrutiny as markers of AD with loss of both structural (Lee et al., 2010; Wang et al., 2015b) and functional (Liu et al., 2014; Wang et al., 2015b) integrity. Here, we observed a similar change in communication pathways, but evidenced by the reduction in direct (metric) connections that characterize functional connectivity between homologous regions in the healthy brain (Lowe et al., 1998; Salvador et al., 2005). Instead communication in AD patients may be facilitated by multiple, indirect paths. However, although the dominant process in neurodegeneration is a loss of functional connectivity, more circuitous paths may in fact serve to increase signal noise, leading to reductions in cognitive performance. Solving the direction of this effect will require longitudinal studies where the change in cognitive performance can be related to characterizations of the neuroimaging, including semi-metric analyzes.

Previous studies on the function connectome of AD patients have noted decreased inter-regional connectivity, but increased intra-regional connectivity, that has been suggested to occur as part of a compensatory mechanism (Sanz-Arigita et al., 2010; Wang et al., 2015b) or due to pathologies affecting functional connectivity that vary according to distance (Sanz-Arigita et al., 2010). Comparison of parameters from the functional connectome constructed from only those edges associated with the highest correlations have similarly been characterized by the heterogeneity of their results (Tijms et al., 2013). Nevertheless, there is support from these studies for a disrupted connectivity model, particularly at long distances and on highly connected hubs (Buckner et al., 2009) that occurs before the manifestation of clinical symptoms (Brier et al., 2014). This study provides weak, qualitative evidence (Figure 1D) for similar changes to the topology of the functional connection, with increased SMP in AD at shorter mean geometric edge lengths, switching at longer lengths to decreases in SMP.

### Increased Heterogeneity of the Connectome

Accompanying the increase of semi-metric connections globally was greater variance in the topology of the semi-metric sub-graphs of AD patients, such that the requirement that >90% of the group have an indirect connection between any two brain regions is not as frequently satisfied in AD patients, and the backbone is thus sparser (Figure 1B). This aligns with the reduced variation of the correlation coefficients (Figure 1A), suggesting that semi-metric behavior is related to this property of the complex distribution. Variance is a feature of functional connectomes, or indeed of other biomarkers of AD, that is not often reported. Two examples of increased variance in AD include hippocampal mircrostructure, measured with high-field MRI microscopy, that was observed without between-group differences (Antharam et al., 2012), and amyloid deposition in older patients with mild cognitive impairment (Hedden et al., 2009). Amyloid deposition has been linked with changes to functional connectivity prior to the loss of cognitive function (Vemuri et al., 2012). However, more recently the relationship between insoluble amyloid proteins and neurodegeneration has been questioned by the observation that loss of cortical volume can occur without amyloid accumulation in cognitively normal older individuals, although the impact of neurodegeneration on cognitive performance is greater in those with higher levels of amyloid (Wirth et al., 2013). This points to a possible upstream effect of amyloid on atrophy, or that there are likely to be similar or even greater effects of tau pathology on connectivity, but these have not yet been assessed. Variation between individuals in localized pathological changes may have profound effects on direct functional connections between regions, and could therefore account for the inhomogeneity of results between studies.

## Conclusion

AD has been described as a disconnection syndrome (Delbeuck et al., 2003), and the available evidence from this study and others deploying functional connectivity would support that notion. Reductions in connections, particularly at long-ranges are a consistent feature of the disorder with local (within-lobe) increases in connectivity more speculatively explained as a compensatory reaction to the disease.

Through an approach that includes all positive connectivities, this study suggests a refinement of the disconnectivity model, in that compensation for the loss of direct connections occurs at large spatial scales through a proliferation of alternative, indirect pathways in an attempt maintain information transfer. The presence of connectivity changes whilst participants retain some degree of cognitive function suggests that this is at least partially successful during the initial stages of the disease. Furthermore, the significant between-subject variance in the topology of these circuitous paths in AD patients is an indication that this process is independent of the location within the brain of the loss of connectivity.

This greater variance may also go some way to explaining the heterogeneity of results that exclude weak links in their models of connectivity. Variable changes in local microstrucuture could alter the topology of the functional connectome based on strong links alone on an individual-by-individual basis. In turn, this may lead to a wider variety of characteristic parameters than in the healthy brain.

This study is based on a small sample size and therefore caution is needed when interpreting the results. Although head motion was non-significant it was greater in AD patients. Whilst the preprocessing steps ameliorated the effects of the motion, the small sample size limited what additional statistical modelling could be undertaken to further regress out its effect, and thus its contribution to differences in SMP cannot be ruled out. Nevertheless, this study motivates the consideration of weak links as important components of understanding the functional connectome in neurodegenerative disorders.

## Conflict of Interest Statement

The authors declare that the research was conducted in the absence of any commercial or financial relationships that could be construed as a potential conflict of interest.

## Footnotes

^1^http://fsl.fmrib.ox.ac.uk/fsl

^2^http://www.brainwavelet.org

^3^http://fsl.fmrib.ox.ac.uk/fsl/fsl4.0/fslview/atlas-descriptions.html

## Supplementary Material

Supplementary Figure 1 **Regions of significant decrease in grey matter volume in AD patients relative to controls, at a statistical threshold at the cluster level of *p* < 0.001 uncorrected**.
