# Supplementary figures and images for: A Winding Road: Alzheimer’s Disease Increases Circuitous Functional Connectivity Pathways
Source: Front Comput Neurosci. 2015 Nov 18;9:140. doi: 10.3389/fncom.2015.00140 (PMC4649041; doi:10.3389/fncom.2015.00140)

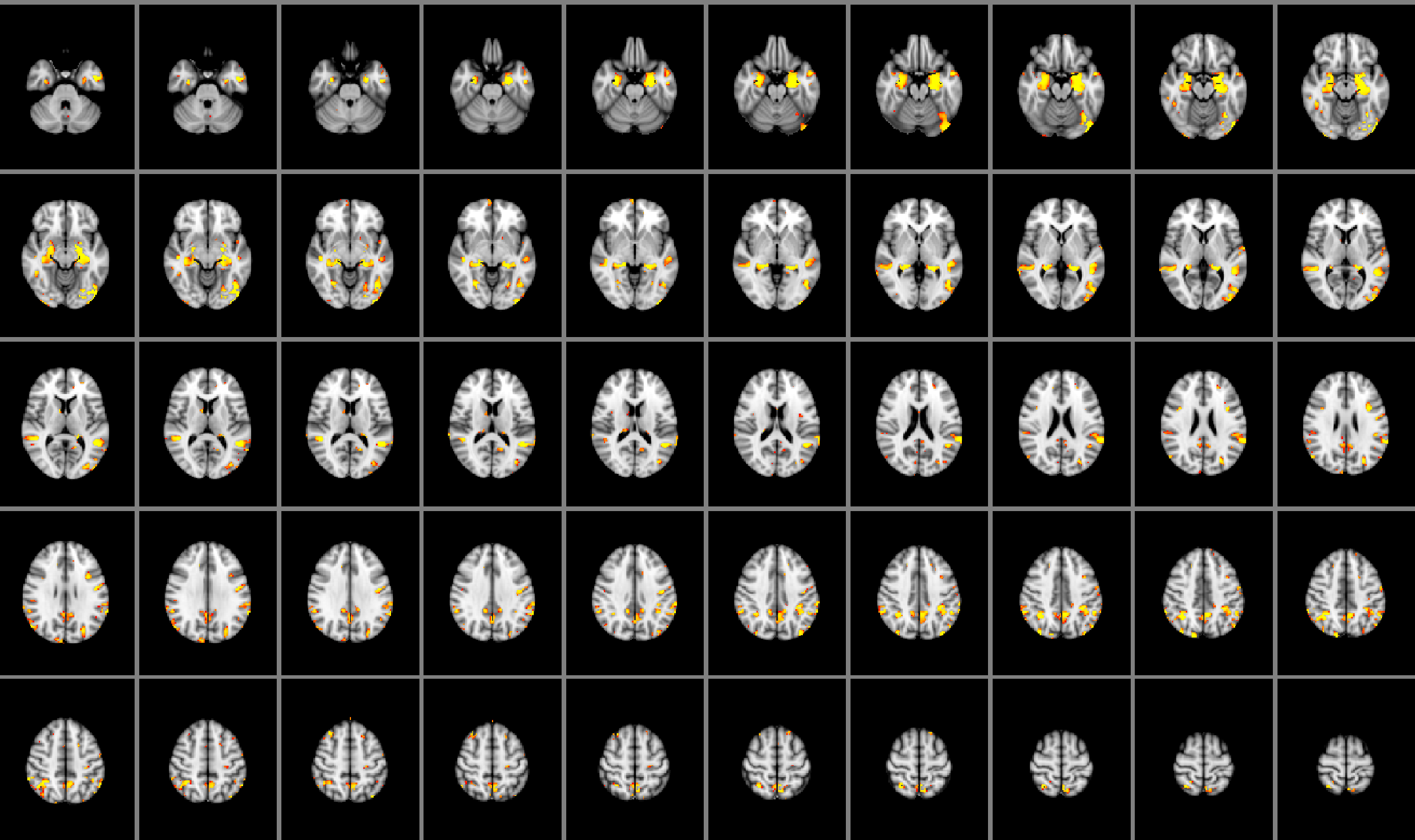

Supplement: Supplementary Figure 1 — Regions of significant decrease in grey matter volume in AD patients relative to controls, at a statistical threshold at the cluster level of p < 0.001 uncorrected. [file Image_1.tif]
